# Burden of respiratory viral infection in persons with human immunodeficiency virus

**DOI:** 10.1111/irv.12734

**Published:** 2020-03-09

**Authors:** Subhashini A. Sellers, Kenton L. Dover, Aubrey G. Bailey, Avery Cheves, Anthony B. Eason, Elena B. Popowitch, Melissa B. Miller, David A. Wohl, Dirk P. Dittmer, William A. Fischer

**Affiliations:** ^1^ Division of Pulmonary and Critical Care Medicine The University of North Carolina at Chapel Hill Chapel Hill North Carolina; ^2^ Department of Microbiology and Immunology The University of North Carolina at Chapel Hill Chapel Hill North Carolina; ^3^ UNC Hospitals McClendon Clinical Laboratories Chapel Hill North Carolina; ^4^ Department of Pathology and Laboratory Medicine The University of North Carolina at Chapel Hill Chapel Hill North Carolina; ^5^ Division of Infectious Diseases The University of North Carolina at Chapel Hill Chapel Hill North Carolina

**Keywords:** acquired immune deficiency syndrome, human immunodeficiency virus, next‐generation sequencing, respiratory virus, viral pneumonia

## Abstract

This study was conducted to determine the prevalence of respiratory viral infections (RVI) in persons living with HIV (PLH) admitted with a respiratory complaint using real‐time reverse transcription polymerase chain reaction and primer‐independent next‐generation sequencing (NGS). Of 82 subjects, respiratory viruses were the most common pathogen identified in 27 (33%), followed by fungus and bacteria in 8 (10%) and 4 (5%) subjects, respectively. Among subjects with RVI, 11 (41%) required ICU admission and 16 (59%) required mechanical ventilation. The proportion of respiratory viruses identified, and the associated complicated hospital course highlights the significant role that RVIs play in the lung health of PLH.

## 1 INTRODUCTION

Pulmonary complications, including respiratory tract infections, are a major cause of morbidity and mortality among people living with HIV (PLH).^1^, ^2^ While bacterial and fungal pathogens have been well ‐described as important etiologies of opportunistic lung disease, less is known about respiratory viruses in PLH.^3^ Studies from antiretroviral therapy (ART)‐limited regions revealed a 20‐fold higher risk of respiratory viral infections among pediatric and adult PLH compared with HIV‐uninfected subjects.^4^, ^5^ In these settings, HIV infection was also a risk factor for higher rates of hospitalization, pneumococcal coinfection, and the need for supplemental oxygen in lower respiratory tract influenza infection.^5^, ^6^, ^7^ The aim of our study was to determine the prevalence of respiratory viruses in a cohort of PLH hospitalized with a respiratory complaint at a tertiary care center. Secondarily, commercial real‐time reverse transcription polymerase chain reaction (rRT‐PCR) was compared to pathogen‐agnostic next‐generation sequencing (NGS) for the detection of viral pathogens in respiratory specimens.

## 2 MATERIALS AND METHODS

This prospective, observational study was conducted at a tertiary care center where over 1800 PLH receive care. The population is 70% male and 60% black, which is reflective of PLH in the United States. PLH ≥ 18 years of age requiring admission between 08/2015 and 08/2018 were approached for enrollment sequentially on weekdays if they had a respiratory symptom (predefined as cough, shortness of breath, sore throat, rhinorrhea, wheezing, or stridor) documented as new and present on admission. Informed consent was obtained from all subjects. This study was approved by the institutional review board at the University of North Carolina.

Clinical information including demographics, presenting symptoms, laboratory chemistries and hematology, medical history, medications, CD4 cell count and HIV plasma viral load, utilization of supplemental oxygen and critical care resources, microbiologic results, radiographic evaluation, and clinical outcomes of hospital course were collected by chart review.

Nasopharyngeal (NP) swabs for each patient were collected in viral transport media and stored at −80°C within 24 hours of enrollment. Excess sample from bronchoalveolar lavage was collected and stored at −80°C if a clinically indicated bronchoscopy was performed. The eSensor RVP (GenMark) rRT‐PCR platform was used to detect influenza A, influenza A H1, influenza A H3, influenza A 2009 H1N1, influenza B, respiratory syncytial virus A, respiratory syncytial virus B, parainfluenza virus 1, parainfluenza virus 2, parainfluenza virus 3, parainfluenza virus 4, human metapneumovirus, human rhinovirus, and coronavirus (229E, HKU1, NL63, OC43). Non‐viral microbiologic diagnoses were based on testing ordered by the healthcare provider and included bacterial, fungal, and AFB cultures, and direct fluorescence antibody (DFA) for *Pneumocystis jirovecii* pneumonia (PJP) from respiratory samples.

Samples collected from individuals enrolled between August 2015 and August 2016 were also tested for RNA respiratory viruses using primer‐agnostic next‐generation deep genome sequencing (Supplemental Methods).

## 3 RESULTS

### 3.1 Screening/enrollment

Of 101 subjects that met criteria for enrollment, 12 refused participation, four were discharged prior to recruitment, two were Spanish‐speaking only, and one was decisionally impaired with no available legally authorized representative.

Eighty‐two subjects including 49 (60%) men with a mean age of 50 were included in the final analysis (Table 1). Demographic, clinical characteristics, and outcomes were similar between those with and without a respiratory virus with the exception of a significantly higher BMI in the group with a virus. Approximately 1/3 each were current or former smokers, and less than half received an influenza vaccine in the prior season. While more than 70% were prescribed antiretroviral therapy, 39% had plasma HIV RNA levels that were above the limits of detection. More than half of the participants had CD4 cell counts below 200/mm^3^.

**Table 1:** Demographics, clinical characteristics, and outcomes of PLH admitted To a US tertiary care center with a respiratory complaint over 3 y, n = 82

Variables	Total subjects (n = 82)	Subjects positive for respiratory virus (n = 27)	Subjects negative for respiratory virus (n = 55)	*P*‐value
Male gender	49 (60%)	14 (52%)	35 (64%)	0.31
Age, mean (SD)	50 (3.41)	51 (2.84)	49.1 (1.94)	0.51
Race				0.38
Caucasian	24 (30%)	6 (23%)	18 (33%)	
African American	55 (68%)	20 (77%)	35 (64%)	
Other	2 (2%)	0	2 (4%)	
Missing	1 (1%)	1 (6%)	0	
BMI, mean (SD)	26 (0.84)	29 (1.82)	24 (0.85)	0.04*
Received influenza vaccine during previous season	38 (46%)	14 (52%)	24 (44%)	0.73
Comorbidities present				
Chronic pulmonary disease	33 (40%)	15 (56%)	18 (33%)	0.05
Cardiac disease	18 (22%)	3 (11%)	15 (28%)	0.09
Liver disease	15 (18%)	6 (22%)	9 (16%)	0.52
Renal disease	29 (36%)	8 (30%)	21 (39%)	0.41
Malignancy	20 (25%)	3 (12%)	17 (31%)	0.06
Rheumatologic disease	6 (7%)	2 (7%)	4 (7%)	0.98
Currently smoking	31 (38%)	17 (63%)	14 (25%)	0.001*
Prior or current intravenous drug use	6 (7%)	4 (15%)	2 (4%)	0.19
On ART	60 (73%)	19 (70%)	41 (75%)	0.69
CD4 + T‐cell count^a^				0.25
<50 cells/µL	24 (30%)	7 (26%)	17 (31%)	
51‐200 cells/µL	19 (23%)	4 (15%)	15 (28%)	
>200 cells/µL	38 (47%)	16 (59%)	22 (41%)	
Missing	1 (2%)	0	1 (3%)	
HIV RNA				
Not detected	49 (60%)	17 (63%)	32 (59%)	0.75
Detectable (VL range)	32 (39%) (42‐933,923)	10 (37%) (49‐933,923)	22 (40%) (42‐656,766)	
Missing	1 (2%)	0	1 (3%)	
Lower respiratory tract findings on chest imaging^b^	62/75 (83%)	19/26 (70%)	43/49 (88%)	0.06
Duration of hospital stay, mean days (SD)	11.2 (1.24)	13.0 (3.13)	10.4 (1.05)	0.33
Intensive care unit required	32 (39%)	11 (41%)	21 (38%)	0.82
Discharged to a higher level of care	18 (22%)	7 (26%)	11 (20%)	0.37
Death	8 (10%)	3 (11%)	5 (9%)	0.77
ARDS	9 (11%)	4 (15%)	5 (9%)	0.44
Acute renal failure	15 (18%)	2 (7%)	13 (24%)	0.07
Myocarditis	2 (2%)	1 (4%)	1 (2%)	0.60
Mechanical ventilation	16 (20%)	5 (19%)	11 (20%)	0.87
Number of days, mean (SD)	7.8 (1.51)	10.4 (1.96)	6.7 (1.97)	0.28
Non‐invasive positive pressure ventilation	13 (16%)	6 (23%)	7 (13%)	0.24
Number of days, mean (SD)	1.38 (0.38)	1.83 (0.83)	1 (0)	0.30
Vasopressors	10 (12%)	3 (11%)	7 (13%)	0.83
Number of days, mean (SD)	8.7 (3.1)	15.3 (9.35)	5.9 (1.98)	0.18

Note

Shown are the demographics for the study cohort overall and grouped by those that tested positive for a respiratory virus and those that tested negative for a respiratory virus. *P*‐values were calculated using Student's *t* test for continuous variables and chi‐squared test for categorical variables.

Abbreviations: BMI, body mass index; ART, antiretroviral therapy; ARDS, acute respiratory distress syndrome.

^a^ CD4 counts <10 were considered equal to 10 for analysis.

^b^ Of those who had chest imaging during admission.

* Indicates a *P*‐value < 0.05.

### 3.2 Respiratory virus detection

Thirty‐four subjects (41%) were diagnosed with a respiratory infection including 27 (33%) with a respiratory virus detected alone or in combination with other pathogens—including one subject with two viruses detected simultaneously, three with both *Pneumocystis jirovecii* pneumonia (PJP) and viral coinfection, and 2 (2%) with bacterial/viral coinfection (Figure S1). Of the seven remaining subjects, PJP was detected in 5 and bacterial pneumonia in 2. Rhinovirus was the most common respiratory viral pathogen detected in 14 subjects (52%), followed by influenza (19%), parainfluenza (11%), coronavirus (7%), and adenovirus (7%). Five respiratory viral infections were identified retrospectively through rRT‐PCR testing conducted as part of this study (Figure S1 **)**.

For 29 participants, 39 respiratory samples (29 NP and 10 BAL) were also tested for RNA viruses using NGS (Figure S2). Three participants had discordant results compared with rRT‐PCR testing: One had adenovirus detected by rRT‐PCR but not by RNA‐based NGS, and two subjects had coronavirus and parainfluenza 4 detected by rRT‐PCR but no virus was identified by NGS. NGS did not detect any additional viruses compared to rRT‐PCR; however, complete genome sequences were obtained for rhinovirus in three subjects that were genetically distinct from all known rhinoviruses (Figure S3).

### 3.3 Clinical outcomes

PLH admitted with a respiratory virus required a high level of care with 41% admitted to the intensive care (ICU) unit and 59% requiring mechanical ventilation. Four subjects with a respiratory virus (15%) were discharged to a higher level of care than on admission, and 3 (11%) died (**Table** **1**). Subjects who died during hospital admission had a lower mean CD4 (43 cells/µL) and higher HIV viral load (165 962 copies/mL) compared to those who were discharged home or to a rehabilitation facility, but pairwise comparisons were not statistically significant (Table S1 **)**.

## 4 DISCUSSION

In this cohort of 82 PLH admitted with a respiratory complaint, respiratory viruses were the most commonly identified respiratory pathogens, more than bacterial and fungal pathogens combined.

Among PLH enrolled in this study, those diagnosed with a respiratory virus were found to have increased BMI (29 vs 24, *P* = .04) compared with those without a respiratory virus. In addition to the fact that increased BMI is a known risk factors for complicated influenza infection in the general population, this suggests that interventions targeting weight loss could reduce risk among PLH.^8^ Similarly, current smoking was significantly more prevalent in those diagnosed with a respiratory virus compared to those without (63% vs 25%, *P* = .001). This mirrors the association between smoking and influenza infection seen in the general population and further supports recommendations for tobacco cessation in this population.^9^ Since HIV‐uninfected individuals were not simultaneously enrolled, it is not possible to determine whether HIV itself is a risk factor for severe disease; however, the high rates of subjects requiring ICU admission (41%), mechanical ventilation (59%), and discharge to a higher level of care than on admission (15%) highlight the severity of illness associated with respiratory viral infections in PLH. These findings support prior studies in resource‐limited settings or that included subjects with poor HIV control found higher rates of hospitalization, longer lengths of stay, and increased need for supplemental oxygen in PLH compared to HIV‐uninfected subjects with viral infection.^5^, ^6^, ^7^ These findings combined with the low rates of influenza vaccination—only 25% of subjects’ influenza were vaccinated, and overall cohort had a 46% vaccination rate—indicate a missed opportunity to reduce morbidity and mortality among PLH.

A substantial proportion of those admitted with a respiratory infection had suboptimally controlled HIV infection, and we are unable to determine whether this was due to recent HIV diagnosis, non‐adherence, or another cause. However, the high burden of respiratory viral infections and marked severity of illness occurred across a wide range of HIV control, including 17 (63%) subjects with undetectable HIV viral loads. While the most severe disease was more common in subjects with lower CD4 counts and higher viral loads, 6/11 (55%) of subjects requiring ICU level care had an undetectable HIV viral load. Understanding the mechanisms that drive the severity of illness associated with respiratory viral infections in people living with HIV will be important to develop more effective therapeutic strategies.

The rate of respiratory viral infections among PLH found in this study also argues for routine respiratory viral diagnostics in the care of PLH with respiratory complaints. Of the 27 subjects diagnosed with a respiratory viral infection, four were identified retrospectively during research testing as part of this study. An undiagnosed viral infection can have significant clinical implications, including unnecessary antibiotic administration and/or the use of therapies that could be harmful in the setting of viral infection such as corticosteroids,^10^, ^11^ in addition to potential nosocomial spread. NGS represents an expansion of diagnostic capacity with the potential to identify viruses not included in the primer‐dependent panel including new or re‐emerging viruses. The ability of NGS to detect genetically distinct rhinoviruses provides proof of principle regarding the utility of NGS to detect viral variants.

Strengths of this study include evaluation of a large cohort of PLH with respiratory symptoms to characterize the burden of respiratory viruses in symptomatic PLH. Combination of both traditional rRT‐PCR and NGS diagnostics also represents a novel and thorough method of characterization. However, there are important limitations that should be considered. Foremost, this study was conducted in a single academic center and was limited to adults. Additionally, our characterization of infectious etiologies was molecular‐based for viruses but relied on less sensitive methods for the detection of bacteria and PJP. However, in support of these findings, viruses were also the most commonly identified pathogen in community‐acquired pneumonia in the EPIC study, which used rigorous microbiologic testing, suggesting that the prevalence of viruses identified in this study is not due to disparate diagnostic sensitivity.^12^ We also cannot exclude that viruses identified could represent asymptomatic carriage in the nasopharynx; however, all subjects were symptomatic on admission, and with the exception of four subjects that had concomitant PCP or bacterial infection, no other respiratory pathogens were identified. Lastly, the use of NGS in this study was exploratory and limited to a subset of participants. The limit of detection by the number of reads being sequenced emerged as a constraint of NGS as seen previously.^13^, ^14^ Likely for this reason, a coronavirus was identified by RT‐PCR, but not by NGS. One obvious solution to overcome this limitation is to sequence to greater depth. Advances in NGS technology since this study make this a cost‐neutral improvement. Another option is the transition to longer read platforms as longer reads are able to detect viruses with less homology to existing genomes, that is, novel pathogens. Lastly, one could add a pre‐selection/ pre‐amplification step, analogous to current, gene‐targeted specialized NGS panels for cancer and other disease. Such a strategy would trade an increase in sensitivity, for the ability of NGS to detect novel respiratory pathogens, that is, genomes that were not included in rRT‐PCR or NGS‐enrichment‐PCR panels.

In conclusion, respiratory viruses were the most common respiratory pathogen identified in admitted PLH. These subjects experienced severe illness as 41% required ICU admission, 59% required mechanical ventilation, and 11% died. Respiratory viral infections represent an important cause of severe illness in PLH admitted with respiratory symptoms. Future research should be directed at understanding if PLH are at increased risk of respiratory viral infections or increased severity compared with HIV‐uninfected individuals in the setting of widespread ART availability and in low prevalence HIV settings, and if so, potential mechanisms underlying that increased risk.

## CONFLICT OF INTEREST

No conflicts of interest to report.

## AUTHOR CONTRIBUTIONS

**Subhashini Sellers:** Data curation, Formal analysis, Investigation, Methodology, Project administration, Writing‐original draft, Writing‐review & editing. **Kenton Dover:** Data curation, Writing‐review & editing. **Aubrey Bailey:** Data curation, Formal analysis, Investigation, Methodology, Software, Visualization, Writing‐original draft, Writing‐review & editing. **Avery Cheves:** Data curation, Formal analysis, Methodology, Validation, Writing‐original draft, Writing‐review & editing. **Anthony Eason:** Data curation, Project administration, Writing‐review & editing. **Elena Popowitch:** Data curation, Formal analysis, Methodology, Validation, Writing‐original draft, Writing‐review & editing. **Melissa Miller:** Conceptualization, Data curation, Formal analysis, Funding acquisition, Investigation, Methodology, Resources, Supervision, Validation, Writing‐review & editing. **David Wohl:** Investigation, Methodology, Resources, Supervision, Writing‐review & editing. **Dirk Dittmer:** Conceptualization, Data curation, Formal analysis, Funding acquisition, Investigation, Methodology, Resources, Software, Supervision, Validation, Visualization, Writing‐original draft, Writing‐review & editing. **William Fischer:** Conceptualization, Data curation, Formal analysis, Funding acquisition, Investigation, Methodology, Project administration, Resources, Supervision, Writing‐original draft, Writing‐review & editing.

## NOTATION OF PRIOR ABSTRACT PRESENTATION

American Thoracic Society Annual Meeting, Washington, DC, May 2018.

## ETHICS COMMITTEE APPROVAL

This study received approval from the University of North Carolina at Chapel Hill IRB, (13‐2140).

## Supporting information

Figure S1

Figure S2

Figure S3

Supplementary Material
