# Supplementary figures and images for: Burden of respiratory viral infection in persons with human immunodeficiency virus
Source: Influenza Other Respir Viruses. 2020 Mar 9;14(4):465–9. doi: 10.1111/irv.12734 (PMC7298306; doi:10.1111/irv.12734)

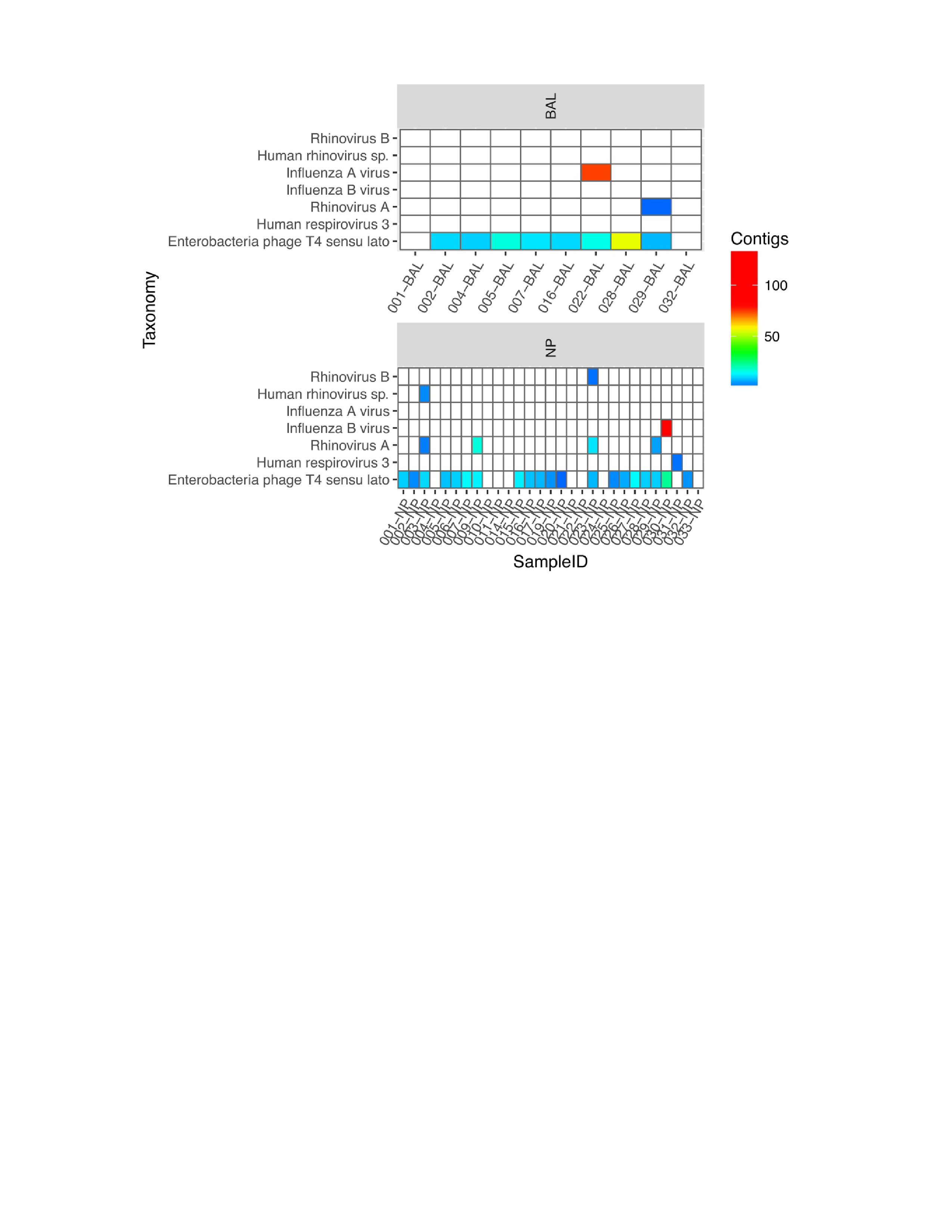

Supplement: Supplementary file 1 — Figure S1 [file IRV-14-465-s001.tiff]

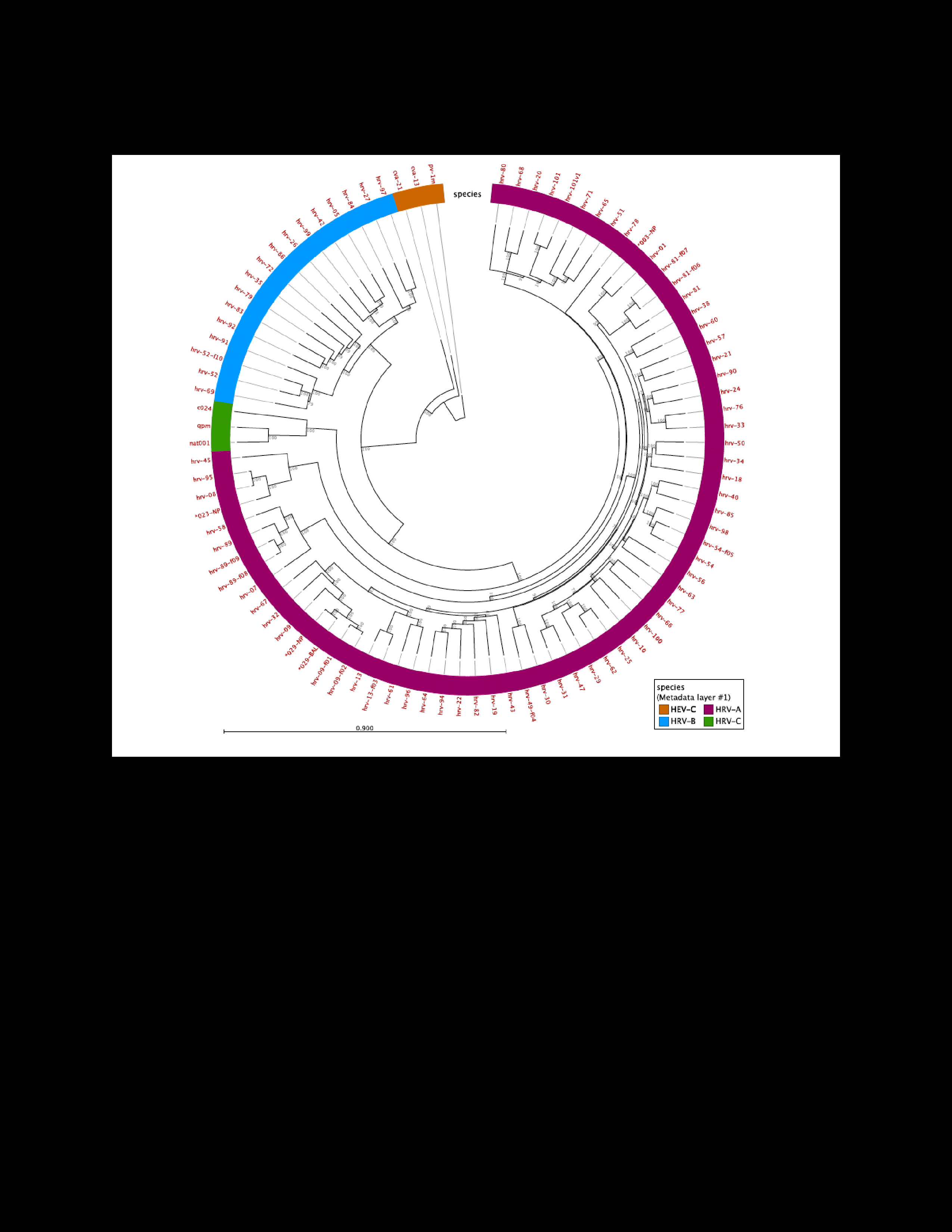

Supplement: Supplementary file 3 — Figure S3 [file IRV-14-465-s003.tiff]
