# Supplementary material for: Burden of respiratory viral infection in persons with human immunodeficiency virus
Source: Influenza Other Respir Viruses. 2020 Mar 9;14(4):465–9. doi: 10.1111/irv.12734 (PMC7298306; doi:10.1111/irv.12734)
Supplement: Supplementary file 4 — Supplementary Material [file IRV-14-465-s004.docx]

**Supplementary Table 1: Characteristics of HIV disease control by outcomes in patients infected with a respiratory virus (n=27)**

|  | Mean CD4 count (cells/uL) | P-value | Mean viral load  (copies/ml) | P-value |
| --- | --- | --- | --- | --- |
| Highest level of care |  | 0.952 |  | 0.008 |
| Floor (n=11) | 527 |  | 93,128 |  |
| Intermediate Care (n=5) | 316 |  | 55,056 |  |
| ICU (n=11) | 259 |  | 49,920 |  |
| Mechanical ventilation |  | 0.120 |  | 0.652 |
| Mechanical ventilation (n=14) | 66 |  | 104,457 |  |
| No mechanical ventilation (n=4) | 425 |  | 60,297 |  |
| Discharge destination |  | 0.048 |  | 0.002 |
| Died during admission (n=3) | 43 |  | 165,962 |  |
| Discharged to rehabilitation (n=3) | 575 |  | 6100 |  |
| Discharged home (n=12) | 389 |  | 66,326 |  |

Shown are the mean CD4 count and mean viral load for patients that tested positive for a respiratory virus grouped by outcome.

Description of abbreviations: ICU = intensive care unit

P-values were calculated for groups of two using the Student’s t-test and for groups of three using ANOVA. Pairwise analysis of means using Bonferroni method for post-hoc analysis of significant ANOVA values demonstrated no significant comparisons.

**Supplementary Methods**

Next Generation Sequencing

RNA from NP swabs and BALs from individuals enrolled from 8/2015 to 8/2016 were extracted using Qiagen QIAamp Viral RNA Mini Kit (Qiagen, Hilden, Germany). Libraries were prepared using Life Technologies Ion Total RNA seq kit v2 with the following modifications to manufacturer’s procedures: 10 µl RNA was input into the fragmentation reaction with 10-minute incubation at 37°C. All 12 µl of fragmented RNA was input into the next step.  Ligation was for 1 hour. Libraries were barcoded and amplified for 16 cycles. After purification according to protocol, one additional bead clean-up was performed. Libraries were sequenced on an Ion Torrent S5 sequencer.

**Bioinformatics.** The virus discovery pipeline follows the concept of digital subtractive hybridization^12,13^. Adaptors were trimmed using Ion Torrent Suite v5.2.2 and errors corrected using BBduk v37.00. FASTQ files were aligned to the human genome build hg38 using BBMap v37.00 and only unaligned reads were kept. Ribosomal sequences were removed using BBMap against and arb-silva.de, release 128 (‘usemodulo=t’ parameter. Duplicate sequences were removed using dedupe.sh v37.00 and the reads *de novo* assembled using the MEGAHIT v1.1.1^14^. Contigs that contained at least two independent reads were queried against the NCBI nt database using blastn. The top hit for each contig was retained. Further analyses were conducted in R v3.3 and custom scripts. Code is available at bitbucket.org/dittmerlab/metagenomic-ggplot-heatmap. Human-filtered sequences were deposited BioProject accession PRJNA401429.

**Figure Legends:**

**Supplementary Figure 1a and 1b: Pathogens detected in the respiratory samples of PLH admitted with respiratory symptoms to a US tertiary care center**

**Supplementary Figure 2: Heatmap depicting viruses identified by NGS in respiratory samples.**

The vertical axis shows the taxa that were detected in at least one sample, the horizontal axis shows the sample ID in alphanumerical order. The color indicates the number of contiguous sequences (contigs) with length ≥ 300 and comprising at least 2 reads for each combination.

**Supplementary Figure 3: Tree demonstrating the phylogenetic relationship between known rhinovirus sequences and the novel strain identified.** A neighbor-joining (NJ) phylogenetic tree showing relationships between all known human rhinovirus (HRV) strains created on the basis of full genome sequences. The human enterovirus C (HEV-C) sequences (poliovirus 1M, coxsackievirus a13, and coxsackievirus a21) were used as outgroups. Each internal node on this tree is annotated with NJ bootstrap values (percentage of 1000 sampled trees). Branch lengths are proportional to the computed pair-wise distance for each sample. The three newly assembled rhinovirus strains are denoted by the sample ID and respiratory tract location from which they were identified. All novel strains are likely members of clade-A, based on genomic similarities.
